# Evaluation of toxoplasma, rubella, and cytomegalovirus seroprevalence and avidity testing in pregnant women

**DOI:** 10.12669/pjms.41.9.12453

**Published:** 2025-09

**Authors:** Banu Humeyra Keskin, Betul Altıntas Oner

**Affiliations:** 1 Banu Humeyra Keskin Zonguldak Obstetrics and Gynecology Hospital, Medical Microbiology, Zonguldak, Türkiye. Current Affiliation: Department of Medical Microbiology, Düzce University Faculty of Medicine, Düzce, Türkiye; 2 Betul Altıntas Oner Eskisehir City Hospital, Infectious Diseases and Clinical Microbiology, Eskişehir, Türkiye

**Keywords:** Congenital infection, Cytomegalovirus, Toxoplasma, Pregnancy, Rubella

## Abstract

**Objectives:**

This study aimed to determine the seroprevalence of Toxoplasma, Rubella, and Cytomegalovirus (CMV) pathogens capable of transplacental transmission leading to significant perinatal morbidity and mortality in pregnant women, and to provide updated regional data for clinical guidance.

**Methodology:**

In this retrospectively study toxoplasma, rubella and CMV antibody results studied using electrochemiluminescence immunoassay in the sera of pregnant women who applied to Zonguldak Gynecology and Obstetrics Hospital between June 2022 and June 2024 were retrospectively analyzed.

**Results:**

Among 2816 pregnant women, IgG seropositivity was observed in 697 (24.8%) for *Toxoplasma gondii*, 2623 (93.6%) for Rubella, and 2755 (98.3%) for CMV. IgM seropositivity was found in 62 (2.2%), 18 (0.6%), and 21 (0.7%) patients, respectively. Toxoplasma IgG seropositivity was significantly higher in women aged ≥40 years, while Rubella IgG positivity peaked in the 25–29 age group (p<0.05).

**Conclusion:**

In conclusion, the rate of Toxoplasma seronegativity found in our study suggests that screening of women would be beneficial and that studies of these infections, in which regional differences in seroprevalence are observed, would contribute to the development of appropriate strategies for screening.

## INTRODUCTION

TORCH infections, including *Toxoplasma gondii*, others (hepatitis viruses, parvovirus, human immunodeficiency virus, Epstein-Barr virus, syphilis), rubella, cytomegalovirus (CMV) and herpes simplex virus (HSV), can cause asymptomatic or mild symptoms in healthy adults and transplacental infection and severe perinatal morbidity and mortality if transmitted during pregnancy.^1^,^2^ Therefore, screening for TORCH group infections during pregnancy, especially in the first trimester, is recommended.^3^,^4^ However, studies show that the management of perinatal infections in the antenatal period is not yet fully standardised.^5^

*Toxoplasma gondii*, an obligate intracellular parasite, can be transmitted to humans by eating raw or undercooked meat containing live tissue cysts, by consuming water or food contaminated with oocysts shed from cat faeces, by vertical transmission during pregnancy, or by tissue, organ or blood transplantation.^6^,^7^ Ninety per cent of babies with congenital toxoplasmosis are asymptomatic in the neonatal period. In later periods, in addition to hydrocephalus, intracranial calcifications and chorioretinitis, cataracts, glaucoma, hepatitis, myositis, myocarditis and mental retardation may cause serious and irreversible consequences.^8^

Rubella virus is a single-stranded RNA virus and 20-50% of patients with this infection have an asymptomatic course.^9^ Congenital rubella syndrome, is caused by infection during the first two months of pregnancy. The syndrome results in multi-organ damage such as congenital heart disease, glaucoma, cataracts, deafness and mental retardation.^10^

CMV is a common herpes virus that often causes asymptomatic infections but can lead to serious complications in immunocompromised patients and those with congenital disease.^11^ Particularly after intrauterine transmission, it can lead to severe congenital malformations, neurological developmental delay and sensorineural hearing loss.^12^

In order to decide whether routine screening for these agents should be performed in a region, the seropositivity rates of that region should be known. The aim of this study was to determine the seroprevalence of toxoplasma, rubella and cytomegalovirus in pregnant women attending the antenatal clinic of our hospital and to contribute to the literature.

## METHODS

Serological test results for *Toxoplasma gondii*, rubella and CMV of 2816 pregnant women admitted to Zonguldak Obstetrics and Gynecology Hospital between June 2022 to June 2024 were retrospectively analyzed. All trimesters included. The patients were divided into five groups according to their age: 18-24 years, 25-29 years, 30-34 years, 35-39 years and over 40 years.

### Ethical approval:

Ethical approval for our study was obtained from the Non-Interventional Clinical Research Ethics Committee of Zonguldak Bülent Ecevit University (Decision No. 2024/13 dated July 10, 2024).

*Toxoplasma gondii*, rubella and CMV antibodies in patient sera sent to the laboratory were analysed by electrochemiluminescence immunoassay (Roche COBAS 6000, Germany) using Elecsys kits. According to the kit instructions, for Toxoplasma IgM, <0.8 COI negative, 0.8-1 COI intermediate, ≥ 1 COI positive; for Toxoplasma IgG, <1 IU/ml negative, 1-3 IU/ml intermediate, ≥ 3 IU/ml positive; for Rubella IgM, <0.8 COI negative, 0. 8-1 COI intermediate, ≥ 1 COI positive; for Rubella IgG, <10 IU/ml negative, ≥ 10 IU/ml positive; for CMV IgM, <0.7 COI negative, 0.7-1 COI intermediate, ≥ 1 COI positive; for CMV IgG, <0. 5 U/ml negative, 0.5-1 U/ml intermediate, ≥ 1 U/ml positive; for CMVIgG, <0.5 U/ml negative, 0.5-1 U/ml intermediate, ≥ 1 U/ml positive. Avidity testing was performed at an external centre and scored as low, intermediate and high avidity.

### Statistical Analysis:

Statistical analysis of data was performed using IBM SPSS Statistics 23. Data were presented as numbers and percentages. Chi-square, Fisher’s exact and Fisher-Freeman-Halton tests were used to analyse the results. p< 0.05 values were considered statistically significant.

## RESULTS

In our study, when the *Toxoplasma gondii*, rubella and CMV test results of 2816 pregnant women admitted to our hospital were retrospectively analyzed, the mean age of the patients was 29.78±5.59 and the age range was 18-48 (min-max).

When all patients were analyzed, IgG antibody results for *Toxoplasma gondii*, Rubella and CMV were positive in 697 (24.8%), 2623 (93.6%) and 2755 (98.3%) patients, respectively, and IgM antibody results were positive in 62 (2.2%), 18 (0.6%) and 21 (0.7%) patients, respectively. The distribution of serological test results by age is shown in Table-I.

**Table-I:** Distribution of toxoplasma, rubella and CMV seroprevalence by age group.

	Toksoplazma		Rubella		CMV	
	IgM n (%)	IgG n (%)	IgM n (%)	IgG n (%)	IgM n (%)	IgG n (%)
***18-24 years (n=514)***						
Negative	494 (96.5)	390 (76.5)	507 (98.8)	38(7.4)	501 (97.9)	10 (2)
Intermediate	6 (1.2)	0 (0)	3 (0.6)	-	8 (1.6)	0 (0)
Positive	12 (2.3)	129 (23.5)	3 (0.6)	473 (92.6)	3 (0.6)	500 (98)
***25-29 years (n=901)***						
Negative	873 (97)	725 (80.8)	893 (99.2)	38 (4.2)	884 (98.1)	18 (2)
Intermediate	6 (0.7)	0 (0)	1 (0.1)	-	8 (0.9)	0 (0)
Positive	21 (2.3)	172 (19.2)	6 (0.7)	**858 (95.8)**	9 (1)	879 (98)
***30-34 years (n=811)***						
Negative	795 (96.8)	602 (74.3)	801 (99.1)	53 (6.6)	789 (97.4)	10 (1.2)
Intermediate	7 (0.9)	1 (0.1)	1 (0.1)	-	16 (2)	0 (0)
Positive	19 (2.3)	207 (25.6)	6 (0.7)	756 (93.4)	5 (0.6)	799 (98.8)
***35-39 years (n=466)***						
Negative	453 (97.2)	315 (67.6)	463 (99.6)	38 (8.2)	454 (97.6)	8 (1.7)
Intermediate	4 (0.9)	0 (0)	0 (0)	-	9 (1.9)	1 (0.2)
Positive	9 (1.9)	151 (32.4)	2 (0.4)	426 (91.8)	2 (0.4)	455 (98.1)
***40 years and over (n=124)***						
Negative	123 (99.2)	76 (61.8)	122 (98.4)	13 (10.6)	121 (97.6)	1 (0.8)
Intermediate	0 (0)	0 (0)	1 (0.8)	-	1 (0.8)	0 (0)
Positive	1 (0.8)	**47 (38.2)**	1 (0.8)	110 (89.4)	2 (1.6)	122 (99.2)
p value	0.896	**<0.001**	0.447	**0.008**	0.477	0.427
***Total (n=2816)***						
Negative	2728 (97)	2108 (75.1)	2786 (99.1)	180 (6.4)	2749 (97.8)	47 (1.7)
Intermediate	23 (0.8)	1 (0)	6 (0.2)	-	42 (1.5)	1 (0.0)
Positive	62 (2.2)	697 (24.8)	18 (0.6)	2623 (93.6)	21 (0.7)	2755 (98.3)
Total	2813 (100)	2806 (100)	2810 (100)	2803 (100)	2812	2803 (100)

When Toxoplasma IgM, Rubella IgM, CMV IgG and IgM antibody test results were compared by age group, no statistically significant difference was found between the groups (p=0.896, p=0.447, p=0.477 and p=0.427, respectively).

When the distribution of Toxoplasma IgG positivity was analysed according to age groups, a statistically significant difference was found between the groups (p<0.001) (Table-I). Toxoplasma IgG positivity in patients aged 40 years and older (38.2%) was significantly higher than that in patients aged 30-34 (25.6%), 25-29 (19.2%) and 18-24 (23.5%) (p=0.003, p<0.001, p=0.003, respectively). In addition, although the positivity rate of patients aged 40 years and over was higher than that of patients aged 35-39 years (32.4%), there was no statistically significant difference between them (p=0.225).

When the distribution of Rubella IgG positivity was analysed according to age groups, a statistically significant difference was found between the groups (p=0.008) (Table-I). This difference between groups was due to the fact that Rubella IgG positivity was statistically significantly higher in the 25-29 age group (95.8%) than in the 35-39 (91.8%) and 40+ (89.4%) age groups (p=0.003 and p=0.003, respectively).

When the avidity test results were examined, low avidity for Toxoplasma gondii was detected in 10 patients (24.4%), intermediate value in five patients (12.2%), and high avidity in 26 patients (63.4%). High avidity was detected in all patients tested for rubella and CMV (Table-II).

**Table-II:** Toxoplasma, rubella and CMV IgG avidity results.

	Toksoplazma n (%)	Rubella n (%)	CMV n (%)
Low avidity	10 (24.4)	0 (0)	0 (0)
Intermediate value	5 (12.2)	0 (0)	0 (0)
High avidity	26 (63.4)	15 (100)	37 (100)
Total	41 (100)	15 (100)	37 (100)

## DISCUSSION

*Toxoplasma gondii*, rubella and CMV cause serious infections, particularly in pregnant women, neonates and immunosuppressed individuals.^13^ It is therefore very important that infections caused by these pathogens are screened before and/or during early pregnancy, diagnosed early and treated if possible.^14^ Serological diagnosis of *Toxoplasma gondii*, rubella and CMV is based on the detection of specific IgM and IgG antibodies.^15^ The need for routine screening for these pathogens in pregnancy is still controversial and approaches vary between countries. In our country, a common approach to the effectiveness of screening for these pathogens has not been established. It is necessary to know the seroprevalence of these three agents in order to prevent both maternal and foetal or neonatal infections.^16^ As toxoplasmosis may present with non-specific clinical symptoms, monitoring with serological tests during pregnancy is important.^17^

In our study, the Toxoplasma IgG and IgM positivity rates were 24.8% and 2.2%, respectively. In a study investigating the seroprevalence of TORCH in women of childbearing age in different countries between 2006 and 2019, including data from our country in 2018, the Toxoplasma IgG positivity rate was 31.4% in Mexico, 56.2% in Brazil, 39.5% in Germany, 22.3% in Poland, 34.7% in Turkey (Izmir) and 0.5% in China.^18^ Toxoplasma IgG and IgM positivity rates in women of childbearing age were 11% and 4%, respectively, in Italy^19^ between 2019-2023 and 26.5% and 0.4% in Iran^20^ between 2018-2020. A review examining Toxoplasma seropositivity in pregnant women in Eastern Mediterranean countries included five studies from Pakistan and found the seropositivity rate to be 29.2%.^21^

In the study analyzing the seroprevalence studies of toxoplasmosis in pregnancy carried out in Turkey in the last 30 years, 58 studies and 256612 test results were included, the regional seroprevalence of Toxoplasma IgG was 59. 43% in the Southeast Anatolian region, 43.95% in the Mediterranean region, 40.89% in the East Anatolian region, 34.85% in the Central Anatolian region, 31.80% in the Black Sea region, 31.21% in the Marmara region and 30.25% in the Aegean region, while Toxoplasma IgM regional seroprevalence was found to be 2.21%, 2.77%, 23%, 1.76%, 1.39%, 1.71% and 5.65%, respectively.^22^ This infection is seen at different rates between countries and even between regions within a country, depending on various factors such as dietary habits, lifestyle, socioeconomic status, and geographical conditions. Similar to other studies, the low seropositivity rates observed in our study suggest that educating pregnant women about the transmission routes of Toxoplasma and infection prevention methods, as well as providing them with prenatal screening, could be beneficial in preventing the risk of congenital toxoplasmosis.

In our country, the rubella virus vaccine is included in our immunization calendar.^16^ Immunisation with rubella virus vaccine, which is a live attenuated vaccine, has been shown to prevent infection and congenital rubella syndrome, one of the most feared complications.^10^ Rubella IgG and IgM positivity in our study were found to be 93.6% and 0.6% while 87% and 3% in Italy^19^, 94.6% and 0.5% in Iran^20^, 16% and 2.5% in Pakistan^23^, respectively. In Turkey, Rubella IgG and IgM positivity rates were 98.3% and 0.7% in Tokat,^24^ 94.2% and 0.5% in Bolu^25^, 91.2% and 0.1% in Diyarbakır^26^, and 93.5% and 0.4% in Istanbul^15^, respectively. As a result of vaccination in our country, seropositivity rates are similarly high in different provinces. These rates vary from country to country due to differences in vaccination schedules.

The guidelines of the German Scientific Medical Societies recommend CMV IgM, IgG and IgG avidity testing from the beginning of pregnancy in all pregnant women and in children under three years of age, not just in pregnant women with suspected CMV infection. However, the American Obstetrics and Gynaecology Association and the Centres for Disease Control and Prevention (CDC) do not recommend routine screening for CMV; instead, they state that it would be beneficial to educate pregnant women about CMV and to perform advanced diagnostic testing in the case of suspicion during routine ultrasound follow-up.^12^

In our study, CMV IgG and IgM rates were 98.3% and 0.7%. In a study conducted in Iran, CMV IgG and IgM rates in women of childbearing age were found to be 99.7% and 0.6%, respectively.^20^ The rate was found to be 97.5% and 12.7%, respectively, among pregnant women in Pakistan.^27^ In another study, CMV IgG positivity rates were found to be 79.9% in Mexico, 98.1% in Brazil, 28.3% in Germany, 68.8% in Poland, 98.4% in Turkey (Izmir) and 91.9% in China.^18^ In our country, CMV IgG and IgM rates in pregnant women or women of childbearing age were found to be 98.2% and 1.6% in Bolu,^25^ 98.7% and 1.5% in Diyarbakır^26^, 99.9% and 0.5% in Kars^28^, and 98.25% and 0.32% in Istanbul,^15^ respectively. In our analysis of data from our country, similarly we also found that this rate is above 95%. However, there are significant differences in rates from country to country. CMV infection is the most common congenital viral infection and it is known that it can infect the fetus during reactivation and reinfection, although rarely. It is therefore important to identify pregnant women in the community who are seronegative and to offer them counselling to protect them from CMV infection.

The fact that the duration of the presence of IgM-type antibodies in serum, which are accepted as indicators of acute infection, varies leads to false-positive results in conditions such as pregnancy and autoimmune diseases, and may be insufficient to differentiate between primary infection, reinfection or reactivation. In these cases, IgG avidity and molecular tests are used for diagnosis.^15^ The detection of low avidity in the IgG avidity test performed after a positive IgM test suggests a recent primary infection, whereas the detection of high avidity suggests a past infection or reactivation.^29^ In our study, low avidity was found in 10 (24.4%) patients and intermediate in five (12.2%) patients for Toxoplasma and avidity test should be performed in case of IgM positivity to differentiate primary and non-primary infections.

## CONCLUSIONS

In view of the Toxoplasma gondii seronegativity rate observed in our study, screening women is considered beneficial. The high rubella positivity rate was considered a successful outcome of the vaccination programmes. Further research into these infections, which exhibit regional variations in seroprevalence, will inform the development of effective screening strategies.
